# Supplementary material for: Polyketide synthase gene clusters in the ericoid mycorrhizal fungus Oidiodendron maius: genome-wide prediction, expression profiling, and functional characterization of knockout mutants
Source: Front Plant Sci. 2026 Sep 16;17:1924430. doi: 10.3389/fpls.2026.1924430 (PMC13623903; doi:10.3389/fpls.2026.1924430)
Supplement: Supplementary file 1 [file SupplementaryFile1.zip › Supplementary Materials/Supplementary Folder 1.zip!/Oidma1_AssemblyScaffolds_Repeatmasked/knownclusterblast/cluster30/jgi.p_Oidma1_168891_mibig_hits.html]

| MIBiG Protein | Description | MIBiG Cluster | MiBiG Product | % ID | % Coverage | BLAST Score | E-value |
| --- | --- | --- | --- | --- | --- | --- | --- |
| AAO65324.1 | putative\_arylamine\_N-acetyltransferase | BGC0000236 | Kinamycin\_biosynthetic\_gene\_cluster | 30 | 94.2372881356 | 98 | 1.5e-20 |
| CCP20052.1 | divN\_protein | BGC0001119 | Divergolide\_biosynthetic\_gene\_cluster | 32 | 47.4576271186 | 75 | 1.4e-13 |
